# Case Report: Analysis of Preserved Umbilical Cord Clarified X-Linked Anhidrotic Ectodermal Dysplasia With Immunodeficiency in Deceased, Undiagnosed Uncles

**DOI:** 10.3389/fimmu.2021.786164

**Published:** 2021-12-22

**Authors:** Satoshi Inaba, Yuta Aizawa, Yuki Miwa, Chihaya Imai, Hidenori Ohnishi, Hirokazu Kanegane, Akihiko Saitoh

**Affiliations:** ^1^ Department of Pediatrics, Niigata University Graduate School of Medical and Dental Sciences, Niigata, Japan; ^2^ Institute for Research Promotion, Niigata University, Niigata, Japan; ^3^ Department of Pediatrics, Graduate School of Medicine, Gifu University, Gifu, Japan; ^4^ Department of Child Health and Development, Graduate School of Medical and Dental Sciences, Tokyo Medical and Dental University (TMDU), Tokyo, Japan

**Keywords:** preserved umbilical cord, hyper IgM syndrome, anhidrotic ectodermal dysplasia with immunodeficiency (EDA-ID), NEMO, case report

## Abstract

Family history is one key in diagnosing inborn errors of immunity (IEI); however, disease status is difficult to determine in deceased relatives. X-linked anhidrotic ectodermal dysplasia with immunodeficiency is one of the hyper IgM syndromes that is caused by a hypomorphic variant in the nuclear factor kappa beta essential modulator. We identified a novel *IKBKG* variant in a 7-month-old boy with pneumococcal rib osteomyelitis and later found that his mother has incontinentia pigmenti. Genetic analysis of preserved umbilical cords revealed the same variant in two of his deceased maternal uncles. Analysis of preserved umbilical cord tissue from deceased relatives can provide important information for diagnosing IEI in their descendants.

## Introduction

Family history is one of the most important items of the 10 Warning Signs of Primary Immunodeficiency Diseases for the prediction of primary immunodeficiency diseases (1), now the term inborn errors of immunity (IEI) is used instead in the International Union of Immunological Societies classification (2). However, IEI is often not diagnosed, even when suspected in deceased relatives, because diagnoses and/or diagnostic tools were not available in previous generations. In Japan, preserved umbilical cords are stored at home as a memento of a birth, and Japanese maternity clinics and hospitals customarily present such tissue as a gift to parents. Diagnostic use of dried umbilical cord has been reported for congenital infections by cytomegalovirus (3) and rubella (4), neonatal enterovirus infection (5), and transient abnormal myelopoiesis (6); however, preserved umbilical cord has never been used to diagnose IEI.

X-linked (XL) anhidrotic ectodermal dysplasia with immunodeficiency (EDA-ID) is a rare IEI. XL-EDA-ID is based on a hypomorphic variant of *IKBKG* (on Xq28), which encodes the nuclear factor kappa beta (NF-κB) essential modulator (NEMO). An *IKBKG* variant is also associated with incontinentia pigmenti (IP) in females. Although the typical variant in IP (deletion of exons 4-10, accounting for >80% of cases) is lethal in males (7), hypomorphic variants can result in surviving males and various clinical phenotypes, including ectodermal dysplasia presenting with aberrant development of hair (hypotrichosis or atrichosis), teeth (hypodontia or anodontia with conical incisors), and eccrine sweat glands (hypohidrosis or anhidrosis), recurrent severe infections, osteopetrosis, lymphedema, and colitis (8–10). Not all of these are relevant; however, several variants have genotype-phenotype correlations (11). Herein, we describe a 7-month-old boy with a novel variant of *IKBKG* and acute rib osteomyelitis caused by *Streptococcus pneumoniae*. Two of his deceased maternal uncles were successfully diagnosed by XL-EDA-ID analysis of their preserved umbilical cords, which had been stored for 40 years.

## Case Report

**Figure 1:**
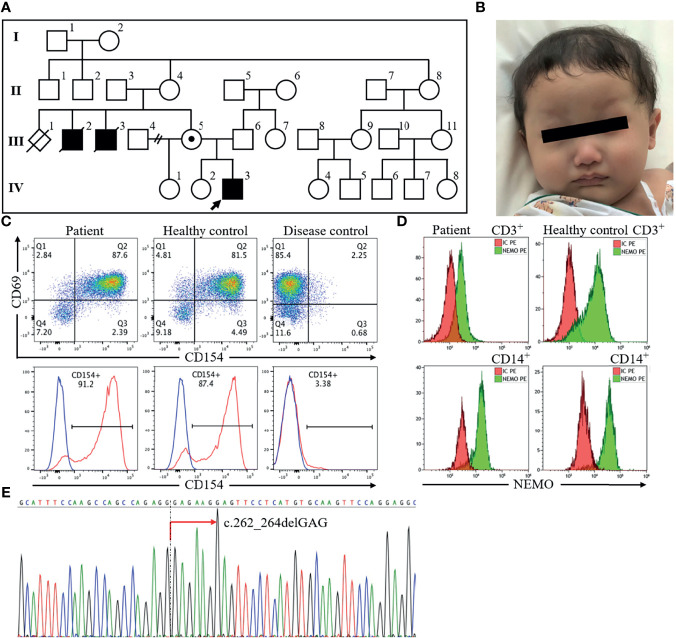
The profile of the index patient. The patient, a 7-month-old boy indicated by an arrow in the pedigree diagram **(A)**, had sparse hair and eyebrows and a depressed nasal bridge **(B)**. Flow cytometric analysis of peripheral blood revealed normal CD40 ligand (CD154) expression **(C)** and decreased NEMO expression in CD3^+^ cells and CD14^+^ cells **(D)**. Sequencing of the *IKBKG* gene revealed hemizygous mutation c.262_264delGAG in exon 3 (red arrow) **(E)**. NEMO, nuclear factor kappa beta essential modulator.

A 7-month-old boy presented to our emergency department with a mass in his left anterior chest and a fever of 2 days’ duration. The patient had delayed umbilical cord separation at 6 weeks of age. He had been vaccinated successfully, without adverse reactions, in accordance with the Japanese national immunization program, and had received one dose of Bacille Calmette-Guérin vaccine and three doses of 13-valent pneumococcal conjugate vaccine, *Haemophilus influenzae* type b vaccine, hepatitis B vaccine, and pentavalent rotavirus vaccine. The patient was born to non-consanguineous parents, and he had been thriving without growth failure. Family history was significant for two maternal uncles who had died at ages 4 and 7 months (**Figure 1A**). The older uncle had persistent refractory diarrhea. The maternal grandmother mentioned that both boys had hypogammaglobulinemia; however, their medical records from 40 years previously had been discarded.

On physical examination, the vital signs were a body temperature of 38.2°C, heart rate of 181 beats/min, respiratory rate of 48 breaths/min, oxygen saturations of 98% in room air, and blood pressure of 123/77 mm Hg. He appeared well. Examination of his left anterior chest wall revealed a firm, non-fluctuant subcutaneous mass, 5 cm in diameter, redness of the overlying skin, and no tenderness. He had dry skin and features of ectodermal dysplasia, including sparse hair and eyebrows, a depressed nasal bridge, no eruption of the deciduous teeth, and decreased sweating indicated by starch-iodine test (12) (**Figure 1B**). Laboratory findings showed an elevated white blood cell count (25,000/μL with 38% polymorphonuclear neutrophils and 55% lymphocytes) and C-reactive protein concentration (7.5 mg/dL). His serum immunoglobulin levels were as follows: IgG 87 mg/dL (reference range: 300-700 mg/dL), IgG1 41.5 mg/dL (136.9-497.8 mg/dL), IgG2 22.4 mg/dL (42.3-159.6 mg/dL), IgA 10 mg/dL (9-55 mg/dL), IgM 148 mg/dL (51-188 mg/dL), and IgE <5.0 IU/mL (≤20 IU/mL). Furthermore, specific antibodies for hepatitis B virus and pertussis-toxin were not detectable despite previous vaccination. His complement components were normal. B lymphocytes were present in peripheral blood (CD19^+^ cells: 17%) (**Supplementary Figure 1**). Chest X-ray findings were normal for the lung and left ribs. Computed tomography of the chest showed that the mass was approximately 2.7 cm in diameter, with poor internal contrast, and that it extended contiguously from the focal osteolytic lesion of the left seventh rib. We started treatment with cefazolin and immunoglobulin replacement for hypogammaglobulinemia. Growth of Gram-positive cocci in chains from three sets of blood culture led to a change in antibiotic from cefazolin to ceftriaxone. His fever resolved and penicillin-susceptible *S. pneumoniae* serotype 6C was isolated. We therefore de-escalated to ampicillin and started trimethoprim-sulfamethoxazole for prophylaxis on the assumption of a diagnosis of hyper IgM syndrome, because hyper IgM syndrome is associated with *Pneumocystis* pneumonia (13). However, the size of the mass did not decrease, and debridement of the abscess and bone curettage were necessary to treat the lesion. Thereafter, the size of the abscess did not increase. Intravenous ampicillin was switched to oral amoxicillin after inflammatory signs improved, and he was discharged on hospital day 51. He completed a 6-month course of antibiotic treatment for chronic rib osteomyelitis with maintenance of IgG trough levels over 700 mg/dL without relapse of the lesion. At 16 months of age, 2 maxillary central conical incisors have erupted.

On the basis of his family history of X-linked recessive form of inheritance and phenotype, X-linked recessive hyper IgM syndrome was suspected. There are two types of X-linked recessive hyper IgM syndrome. Type 1 is the most frequent and is associated with CD40 ligand abnormality. The other type is XL-EDA-ID associated with NEMO abnormality. Flow cytometric analysis of peripheral blood revealed normal expression of CD40 ligand (**Figure 1C**) and decreased NEMO expression in CD3^+^ cells and CD14^+^ cells (**Figure 1D**). Analysis of the *IKBKG* gene by long-range PCR and the Sanger sequence (14) revealed a novel in-frame deletion of a codon (c.262_264delGAG), which resulted in one amino acid microdeletion (∆E88) in exon 3 (**Figure 1E**). The primer pairs and methods for this analysis were shown in **Supplementary Table**.

**Figure 2:**
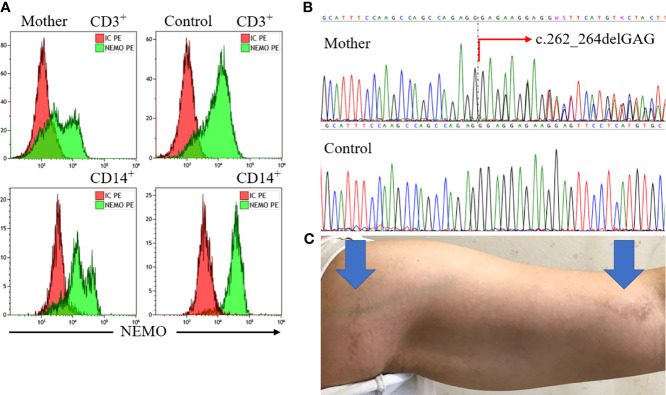
Findings for the mother. Flow cytometric analysis of maternal peripheral blood revealed mixed expression of normal and mutated NEMO **(A)**. Sequencing of the *IKBKG* gene revealed the heterozygous variant c.262_264delGAG (red arrow) **(B)**. The mother’s left arm exhibited linear hyperpigmentation following the Blaschko line (blue arrows) **(C)**. NEMO, nuclear factor kappa beta essential modulator.

**Figure 3:**
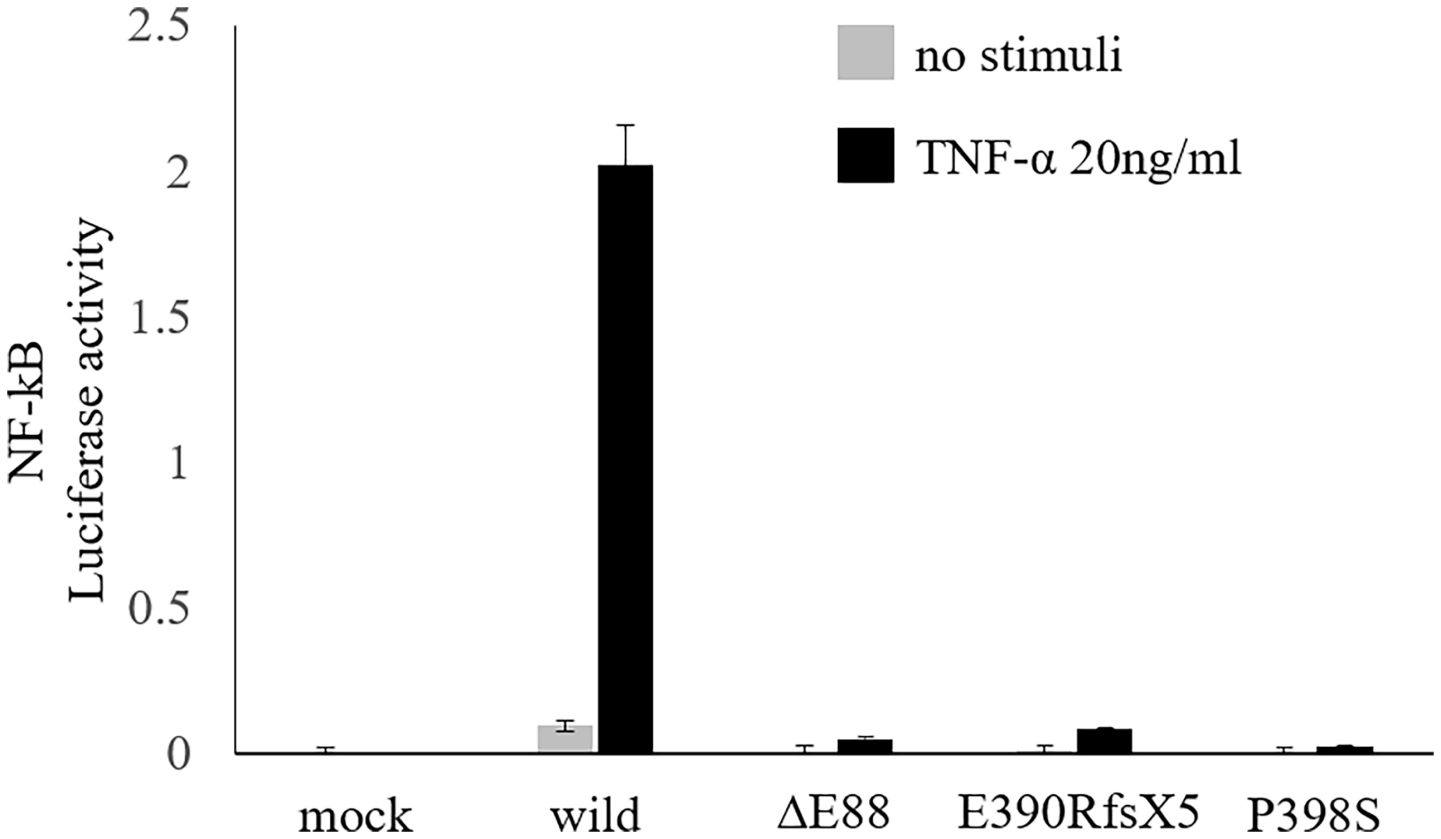
Functional analysis of the variant. NF-κB reporter gene analysis using NEMO-deficient HEK293 cells revealed a loss of activity of the *IKBKG* variant ∆E88, as compared with the wild type, and no response against TNF-α. Hypomorphic variants already reported, i.e., E390RfsX5 and P398S (14), were also included to the analysis to ensure the accuracy of this experiment. Data are shown as the mean and standard error of triplicate measurements. NF-κB, nuclear factor kappa beta; NEMO, nuclear factor kappa beta essential modulator; TNF-α, tumor necrosis factor-alpha.

We further examined his mother and conducted a functional analysis of the variant *in vitro*. Flow cytometric analysis of maternal peripheral blood showed mixed expression of normal and mutated NEMO (**Figure 2A**). The same heterozygous variant (c.262_264delGAG) was also found in the mother (**Figure 2B**). She was ultimately diagnosed with IP on the basis of hypodontia and linear hyperpigmentation that followed the Blaschko line on her left arm (**Figure 2C**). She had no history of suspected immunodeficiency. An NF-κB reporter gene analysis measuring the activity of *IKBKG* variants using NEMO-deficient HEK293 cells (14) showed loss of activity of the *IKBKG* variant ∆E88, as compared with the wild type, and no response against tumor necrosis factor-alpha (TNF-α) (**Figure 3**). Ultimately, we diagnosed XL-EDA-ID due to *IKBKG* ∆E88 in our patient.

**Figure 4:**
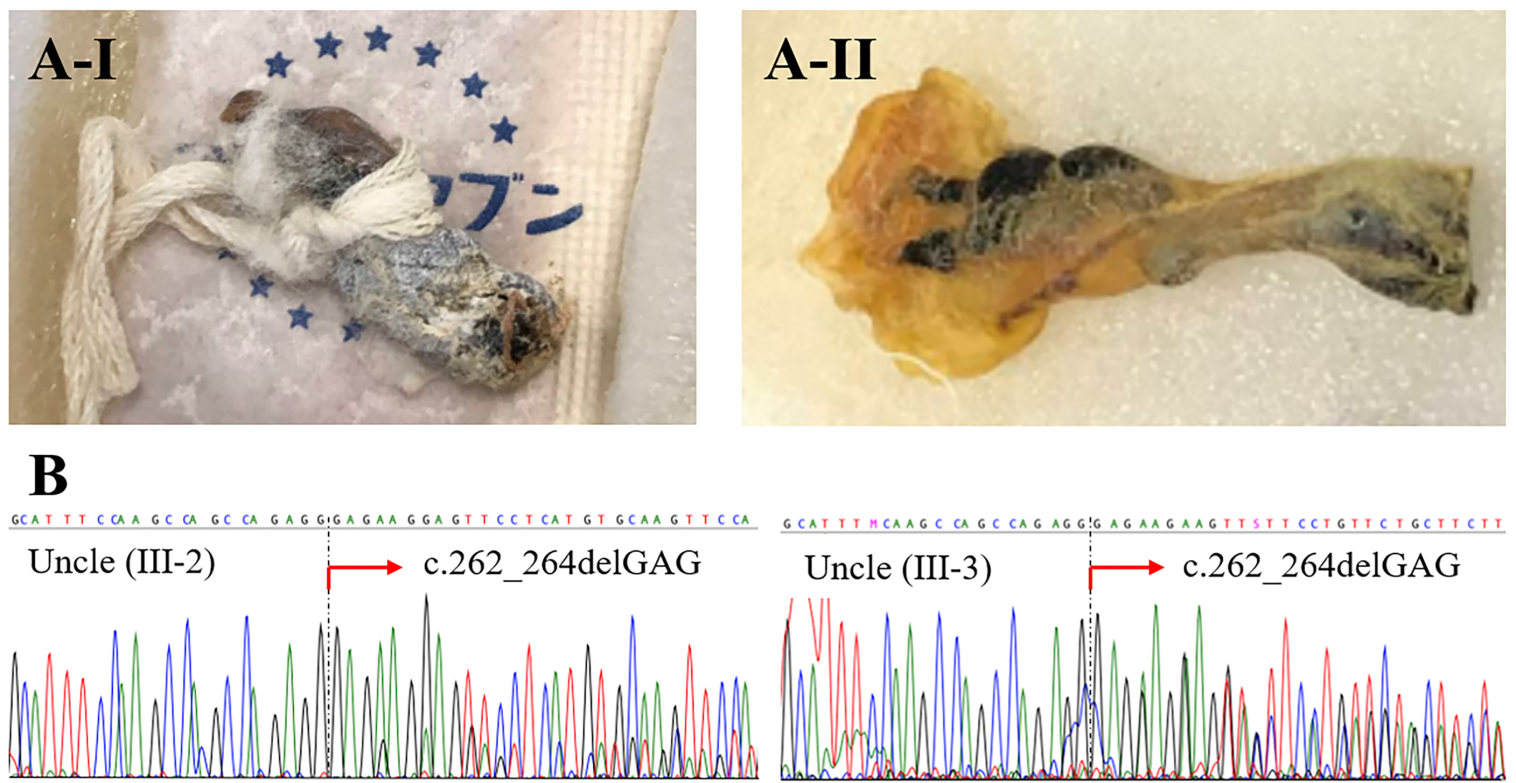
Preserved umbilical cords from deceased relatives. A-I is from the older uncle (Uncle III-2), and A-II is from the younger uncle (Uncle III-3) **(A)**. Sequencing of the *IKBKG* gene revealed the heterozygous variant c.262_264delGAG in both uncles **(B)**.

The family history of infantile death of two maternal uncles suggested that both had XL-EDA-ID. Because their preserved umbilical cords (**Figure 4A**) were available, we extracted DNA with ZR-DuetTM DNA/RNA MiniPrep Plus kit (Zymo Research), in accordance with the manufacturer’s instructions, and performed sequencing with the Sanger sequencing method (**Figure 4B**). These old samples were probably DNA-fragmented by aging; thus, newly designed primers for a shorter target gene of exon 3 of *IKBKG* gene were used instead of long-range PCR (**Supplementary Table**). Although each variant was identified in a heterozygote because of the existence of a pseudogene, both samples were confirmed to be from the uncles by confirming the presence of male-specific *SRY* gene (**Supplementary Table** and **Supplementary Figure 2**). Analysis of their preserved umbilical cords enabled us to diagnose XL-EDA-ID in both maternal uncles. We further examined the maternal grandmother of the index patient. In contrast to the index patient, the mother, and two maternal uncles, sequencing of the *IKBKG* gene using the peripheral blood lymphocytes did not reveal any variants in exon 3 (**Supplementary Figure 3**).

## Discussion

This report used preserved umbilical cord as a tool to diagnose IEI in deceased relatives, after diagnosis of XL-EDA-ID in an infant with a novel variant. Although the maternal uncles died during infancy, the outcomes would likely be different now because of improvements in medical care during the last 40 years, particularly the availability of immunoglobulin products and effective antibiotics. Identification of the same variant in the deceased relatives confirmed the diagnosis and revealed varied phenotypes at the new gene variant site.

Previous reports have described more than 70 *IKBKG* gene variants, but not ∆E88 (15). NEMO is a regulatory protein comprising 419 amino acids and is made up of several domains such as coiled-coil motifs, leucine zipper domain, and zinc finger domain (16). A previous review of phenotypes of individuals with *IKBKG* variants assessed clinical phenotype, infectious susceptibility, and immune capacity and found that several variants have genotype-phenotype correlations (11). Our patient with the ∆E88 variant had many similarities in coiled-coil motif 1, including high susceptibility to polysaccharide encapsulated bacteria due to pneumococcal osteomyelitis and impaired response to TNF-α. However, this is the first report to link this site to hyper IgM and deaths (11, 15). Not all the effects of *IKBKG* variants have been revealed and, similarly, there is no correlation between disease severity and the site of an IP variant in females (17).

When genes of interest have pseudogenes, employing high-throughput sequencing technologies such as targeted gene panels or exome sequencing have difficulty in reliable variant identification (18). In our index patient, the variant was demonstrated by long-range PCR with the removal of the pseudogene and the Sangar sequence (14). The use of next-generation sequencing might have missed the variant.

Although the family history of X-linked recessive form of inheritance and phenotype was a key to diagnose XL-EDA-ID in the index patient, the maternal grandmother did not have the same *IKBKG* variant as the index patient, the mother, and two maternal uncles. This is possibly due to maternal germinal mosaicism, reported in other IEI such as X-linked agammaglobulinemia (19) and X-linked severe combined immunodeficiency (20).

Diagnosis of XL-EDA-ID from preserved umbilical cord tissue was challenging because PCR detection of long nucleotides was not possible, perhaps because of DNA fragmentation. The newly designed primers for shorter target genes, which were based on the index patient’s variant, enabled sequencing of fragmented DNA from preserved umbilical cord. The pseudogene allele might affect the assay, resulting in identification of a gene variant in a heterozygote in males. The hemizygous state for the variant could not be confirmed in the maternal uncles because of this pitfall. Contamination of the umbilical cord by maternal blood was also a possibility; PCR confirmation of the *SRY* gene just demonstrated that the preserved umbilical cord contained male origin tissue.

The analysis of preserved umbilical cord is limited in the areas where it is available; however, the customs of preserving umbilical cord are documented among many countries and cultures outside Japan (4).

In summary, we identified a novel *IKBKG* variant that causes EDA-ID and this is the first to describe the analysis of preserved umbilical cord tissue to diagnose IEI in deceased relatives. When investigating IEI in a family, analysis of preserved umbilical cord tissue from decedents can yield information important for diagnosis of living relatives and help clarify the phenotypes of new gene variant sites.

## Data Availability Statement

The original contributions presented in the study are included in the article/**Supplementary Material**. Further inquiries can be directed to the corresponding author.

## Ethics Statement

The studies involving human participants were reviewed and approved by Gifu University. Written informed consent to participate in this study was provided by the participants’ parents. Written informed consent was obtained from the individual(s), and minor(s)’ legal guardian/next of kin, for the publication of any potentially identifiable images or data included in this article.

## Author Contributions

Patient's management: SI, YA, CI, and AS. Manuscript preparation: SI and YA. Study concept: HK. Study Design: YA, HO, and HK. Literature search: SI, YA, and HK. Data analysis/interpretation: YA, YM, CI, HO, and HK. Manuscript editing: CI, HO, HK, and AS. All authors contributed to the article and approved the submitted version.

## Funding

This work was supported by Niigata Univ. Early-Career Scientists Development Program to YA and by MEXT KAKENHI Grant Number JP21K07770 and Health and Labour Science Research Grants for Research on Intractable Diseases from the Ministry of Health, Labour and Welfare of Japan (Grant Numbers 20316700 and 20317089), and by AMED (Grant Number JP20ek0109480) to HO.

## Conflict of Interest

The authors declare that the research was conducted in the absence of any commercial or financial relationships that could be construed as a potential conflict of interest.

## Publisher’s Note

All claims expressed in this article are solely those of the authors and do not necessarily represent those of their affiliated organizations, or those of the publisher, the editors and the reviewers. Any product that may be evaluated in this article, or claim that may be made by its manufacturer, is not guaranteed or endorsed by the publisher.

## References

[1] Subbarayan A Colarusso G Hughes SM Gennery AR Slatter M Cant AJ. Clinical Features That Identify Children With Primary Immunodeficiency Diseases. Pediatrics (2011) 127:810–6. doi: 10.1542/peds.2010-3680 21482601

[2] Yamashita M Inoue K Okano T Morio T. Inborn Errors of Immunity-Recent Advances in Research on the Pathogenesis. Inflammation Regener (2021) 41:9. doi: 10.1186/s41232-021-00159-6 PMC7992775 33766139

[3] Koyano S Inoue N Nagamori T Yan H Asanuma H Yagyu K. Dried Umbilical Cords in the Retrospective Diagnosis of Congenital Cytomegalovirus Infection as a Cause of Developmental Delays. Clin Infect Dis (2009) 48:e93–5. doi: 10.1086/598506 19351268

[4] Miyata I Kubo T Miyairi I Saitoh A Morimoto N. Successful Detection and Genotyping of Rubella Virus From Preserved Umbilical Cord of Patients With Congenital Rubella Syndrome. Clin Infect Dis (2015) 60:605–7. doi: 10.1093/cid/ciu882 25378458

[5] Miyata I Saitoh A. Detection of Enteroviral RNA From Preserved Umbilical Cord. J Clin Virol (2013) 56:274–5. doi: 10.1016/j.jcv.2012.11.008 23218992

[6] Okamoto T Nagaya K Toriumi N Sarashina T Azuma H. Retrospective Diagnosis of Transient Abnormal Myelopoiesis by Using Preserved Dried Umbilical Cord. Pediatr Int (2021) 63:243–5. doi: 10.1111/ped.14583 34219329

[7] Smahi A Courtois G Vabres P Yamaoka S Heuertz S Munnich A. Genomic Rearrangement in NEMO Impairs NF-kB Activation and is a Cause of Incontinentia Pigmenti. Nature (2000) 405:466–72. doi: 10.1038/35013114 10839543

[8] Zonana J Elder ME Schneider LC Orlow SJ Moss C Golabi M. A Novel X-Linked Disorder of Immune Deficiency and Hypohidrotic Ectodermal Dysplasia Is Allelic to Incontinentia Pigmenti and Due to Mutations in IKK-Gamma (NEMO). Am J Hum Genet (2000) 67:1555–62. doi: 10.1086/316914 PMC1287930 11047757

[9] Döffinger R Smahi A Bessia C Geissmann F Feinberg J Durandy A. X-Linked Anhidrotic Ectodermal Dysplasia With Immunodeficiency is Caused by Impaired NF-kB Signaling. Nat Genet (2001) 27:277–85. doi: 10.1038/85837 11242109

[10] Orange JS Levy O Geha RS. Human Disease Resulting From Gene Mutations That Interfere With Appropriate Nuclear Factor-κb Activation. Immunol Rev (2005) 203:21–37. doi: 10.1111/j.0105-2896.2005.00221.x 15661019

[11] Hanson EP Monaco-Shawver L Solt LA Madge LA Banerjee PP May MJ. Hypomorphic Nuclear Factor-κb Essential Modulator Mutation Database and Reconstitution System Identifies Phenotypic and Immunologic Diversity. J Allergy Clin Immunol (2008) 122:1169–77. doi: 10.1016/j.jaci.2008.08.018 PMC2710968 18851874

[12] Chia KY Tey HL. Approach to Hypohidrosis. J Eur Acad Dermatol Venereol (2013) 27:799–804. doi: 10.1111/jdv.12014 23094789

[13] Toyohara M Kajiho Y Toyofuku E Takahashi C Owada K Kanda S. An Infant With X-Linked Anhidrotic Ectodermal Dysplasia With Immunodeficiency Presenting With *Pneumocystis* Pneumonia: A Case Report. Clin Case Rep (2021) 9:e05093. doi: 10.1002/ccr3.5093 34815879 PMC8593555

[14] Ohnishi H Kishimoto Y Taguchi T Kawamoto N Nakama M Kawai T. Immunodeficiency in Two Female Patients With Incontinentia Pigmenti With Heterozygous NEMO Mutation Diagnosed by LPS Unresponsiveness. J Clin Immunol (2017) 37:529–38. doi: 10.1007/s10875-017-0417-3 28702714

[15] Heller S Kölsch U Magg T Krüger R Scheuern A Schneider H. T Cell Impairment Is Predictive for a Severe Clinical Course in NEMO Deficiency. J Clin Immunol (2020) 40:421–34. doi: 10.1007/s10875-019-00728-y 31965418

[16] Maubach G Schmädicke AC Naumann M. NEMO Links Nuclear Factor-κb to Human Diseases. Trends Mol Med (2017) 23:1138–55. doi: 10.1016/j.molmed.2017.10.004 29128367

[17] Fusco F Bardaro T Fimiani G Mercadante V Miano MG Falco G. Molecular Analysis of the Genetic Defect in a Large Cohort of IP Patients and Identification of Novel NEMO Mutations Interfering With NF-κB Activation. Hum Mol Genet (2004) 13:1763–73. doi: 10.1093/hmg/ddh192 15229184

[18] Claes KB De Leeneer K. Dealing With Pseudogenes in Molecular Diagnostics in the Next-Generation Sequencing Era. Methods Mol Biol (2014) 1167:303–15. doi: 10.1007/978-1-4939-0835-6_21 24823787

[19] Sakamoto M Kanegane H Fujii H Tsukada S Miyawaki T Shinomiya N. Maternal Germinal Mosaicism of X-Linked Agammaglobulinemia. Am J Med Genet (2001) 99:234–7. doi: 10.1002/1096-8628(2001)9999:9999<::aid-ajmg1159>3.0.co;2-m 11241495

[20] O'Marcaigh A Puck JM Pepper AE Santes KD Cowan MJ. Maternal Mosaicism for a Novel Interleukin-2 Receptor Gamma-Chain Mutation Causing X-Linked Severe Combined Immunodeficiency in a Navajo Kindred. J Clin Immunol (1997) 17:29–33. doi: 10.1023/a:1027332327827 9049783

