# Supplementary material for: Case Report: Analysis of Preserved Umbilical Cord Clarified X-Linked Anhidrotic Ectodermal Dysplasia With Immunodeficiency in Deceased, Undiagnosed Uncles
Source: Front Immunol. 2021 Dec 22;12:786164. doi: 10.3389/fimmu.2021.786164 (PMC8727435; doi:10.3389/fimmu.2021.786164)
Supplement: Supplementary file 1 [file DataSheet_1.docx]

Supplementary Material

Supplementary Table. Primer sequences for *IKBKG* and *SRY* genes designed for this study.

|  |  | **Sequences (5' to 3')** |
| --- | --- | --- |
| *IKBKG* |  |  |
| long-range PCR | Forward | GGTGAATTATCAGCATTCTG |
|  | Reverse | AACAGCTGAAGCGTAAGGTG |
| exon2 | Forward | GGTGAATTATCAGCATTCTG |
|  | Reverse | TGAGAGCATGCTAAACTTCC |
| exon3 | Forward | CACTTCTGGCCTCTGACTTC |
|  | Reverse | AGTGTGACGGCTTTTCTGAG |
| exon3 short | Forward | TGCCCAGCTCCCCTCCACTG |
|  | Reverse | CGCAGAGCCTGCTCCTTCTG |
| exon4 | Forward | CTCTTTTTGCCCCATCTGTG |
|  | Reverse | CTATCCTCATCAAGGAGCAC |
| exon5 | Forward | AGTTGTCCTCGTGTCTGTTG |
|  | Reverse | CAAGGCTAAGGGACAGAATG |
| exon6 | Forward | GCTTTAGAGTTGACGGCCTC |
|  | Reverse | CTCCTGAGAGCAACATCCTG |
| exon7 | Forward | GCATCTCCTCTGTCGTTTTG |
|  | Reverse | CTGGGCAACAAGAGCAAAAC |
| exon8 | Forward | CGACGTATCTGTTTCCTCTG |
|  | Reverse | CTAACCCAGAACACCAGGAG |
| exon9 | Forward | TGTTCTGGGTTAGGGCTCAC |
|  | Reverse | CACTCATGTAAGACCAGGAG |
| exon10 | Forward | TGTTGCTCTCTGGAGACTTG |
|  | Reverse | AACAGCTGAAGCGTAAGGTG |
| *SRY* | Forward | ACGCATTCATCGTGTGGTCT |
|  | Reverse | TTCTCTCTGTGCATGGCCTG |

For the analysis of the peripheral bloods, DNA fragments of *IKBKG* gene were amplified by long-range PCR, and then secondary PCR was performed for each exon, and analyzed with the BigDye Terminator v3.1 Cycle Sequencing Kit and an Applied Biosystems 3130xl Genetic Analyzer (Applied Biosystems, Foster City, CA, USA). However, because the amplification of the DNA fragments of *IKBKG* gene by long-range PCR or direct PCR with exon 3 primer sets for the samples derived from the preserved umbilical cords failed, the sequencing of the target site was performed with newly designed primer sets, exon 3 short. DNA fragments were amplified with PCR using KOD FX Neo (TOYOBO, Osaka, Japan). This was performed with an annealing temperature of 62°C at 40 PCR cycles.


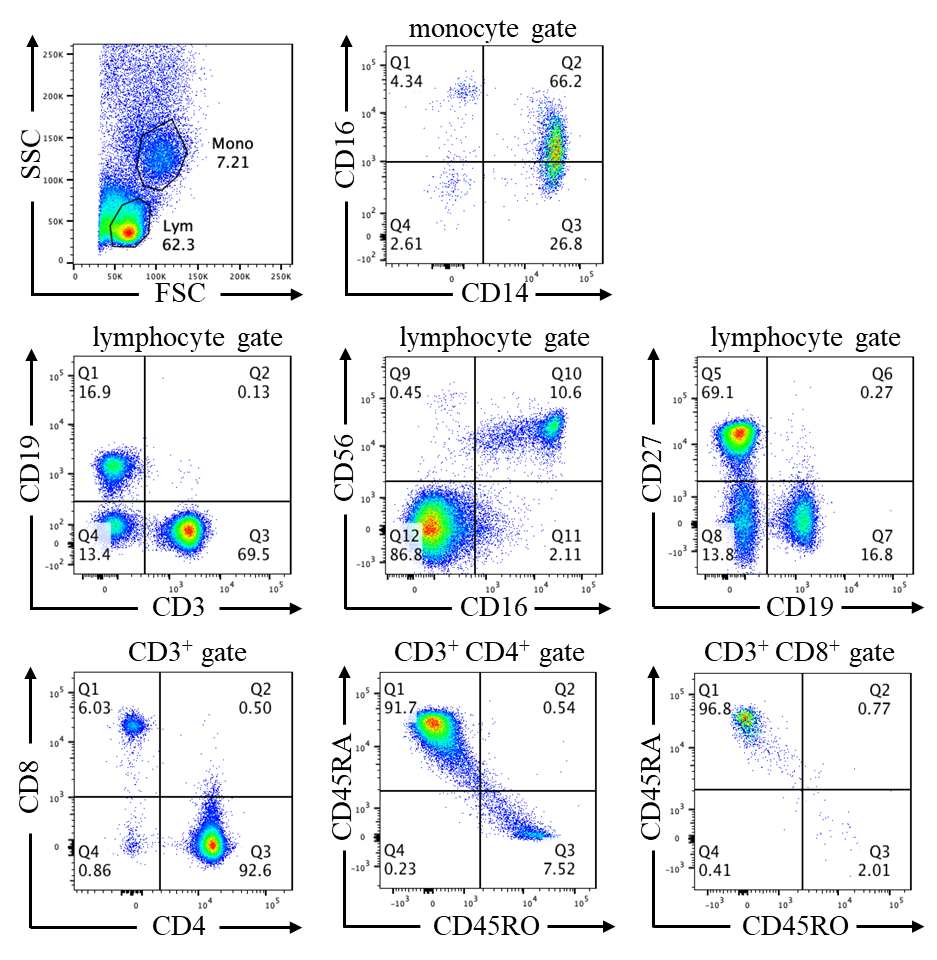


**Supplementary Figure 1.** **Immunological phenotype of the index patient.** We evaluated his immunological phenotype by multicolor flow cytometry (1). Lymphocyte proliferation to PHA was 574.6 SI and to ConA was 242.0 SI.

Abbreviations: SSC, side scatter; FSC, forward scatter; CD, cluster of differentiation; PHA, phytohemagglutinin; Con A, concanavalin A; SI, stimulation index.


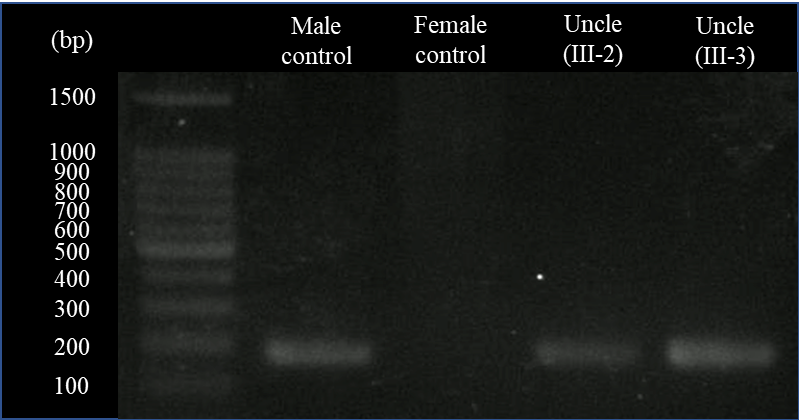


**Supplementary Figure 2.** **Electrophoresis of the *SRY* gene.** The DNA fragments were amplified with PCR using KOD FX Neo (TOYOBO, Osaka, Japan). This was performed with an annealing temperature of 62°C at 40 PCR cycles. The calculated size of the fragments was 175 base pair. Male-specific *SRY* genes were detected in the preserved umbilical cords of two deceased maternal uncles.
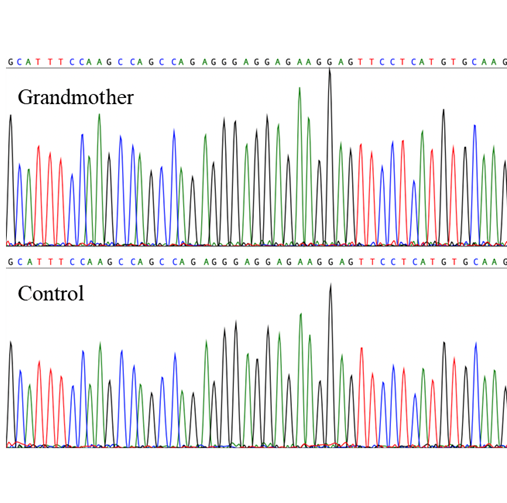


**Supplementary Figure 3.** **Findings for the maternal grandmother.** Sequencing of the *IKBKG* gene using the peripheral blood lymphocytes did not reveal any mutations in exon 3.

# REFERENCES

1. Takashima T, Okamura M, Yeh TW, Okano T, Yamashita M, Tanaka K, et al. Multicolor Flow Cytometry for the Diagnosis of Primary Immunodeficiency Diseases. *J Clin Immunol* (2017) 37:486-495. doi: 10.1007/s10875-017-0405-7.
